# P-506. Spatial Clustering of Congenital Syphilis-Related Stillbirths in Nuevo Leon, Mexico: Identifying Spatial Gaps in Care

**DOI:** 10.1093/ofid/ofaf695.721

**Published:** 2026-01-11

**Authors:** Lindsay Ariadna Concha-Mora, Abril M Gutiérrez-Castro, Paola Quintanilla-Urdiales, Rocio Ximena Sandoval-Orozco, Rubén G Valadez-Mata, Judith Estela Guzman Garcia, Ian Carlo Pineda-Fierro, Jessica Guerra-Díaz, Oscar Tamez-Rivera

**Affiliations:** The Hospital for Sick Children, Toronto, ON, Canada; Pediatric Residency Program, Programa Multicéntrico de Especialidades Médicas ITESM- SSNL, Tecnológico de Monterrey. Escuela de Medicina y Ciencias de la Salud. Monterrey, México, Monterrey, Nuevo Leon, Mexico; Pediatric Residency Program, Programa Multicéntrico de Especialidades Médicas ITESM- SSNL, Tecnológico de Monterrey. Escuela de Medicina y Ciencias de la Salud. Monterrey, México, Monterrey, Nuevo Leon, Mexico; Tecnológico de Monterrey Campus Monterrey, Chihuahua, Chihuahua, Mexico; Hospital Universitario Dr. Jose Eleuterio Gonzalez, Guadalupe, Nuevo Leon, Mexico; Tecnologico de Monterrey, Monterrey, Nuevo Leon, Mexico; Hospital Universitario Dr. Jose Eleuterio Gonzalez, Guadalupe, Nuevo Leon, Mexico; Hospital Universitario Dr. José Eleuterio González, Monterrey, Nuevo Leon, Mexico; Tecnologico de Monterrey, Escuela de Medicina y Ciencias de la Salud, Monterrey, Nuevo Leon, Mexico

## Abstract

**Background:**

Stillbirths due to congenital syphilis are among the most devastating yet preventable outcomes of maternal infection. Vertical transmission remains widespread globally, especially in low-middle-income countries. In Mexico, rising maternal syphilis rates have been accompanied by an increase in CS-related stillbirths, yet the factors driving these outcomes remain unclear. Geographic context—distance to care and local environmental or social conditions—may influence clinical trajectories. Geospatial analysis identifies patterns of vulnerability and informs targeted interventions.

**Image 1.**
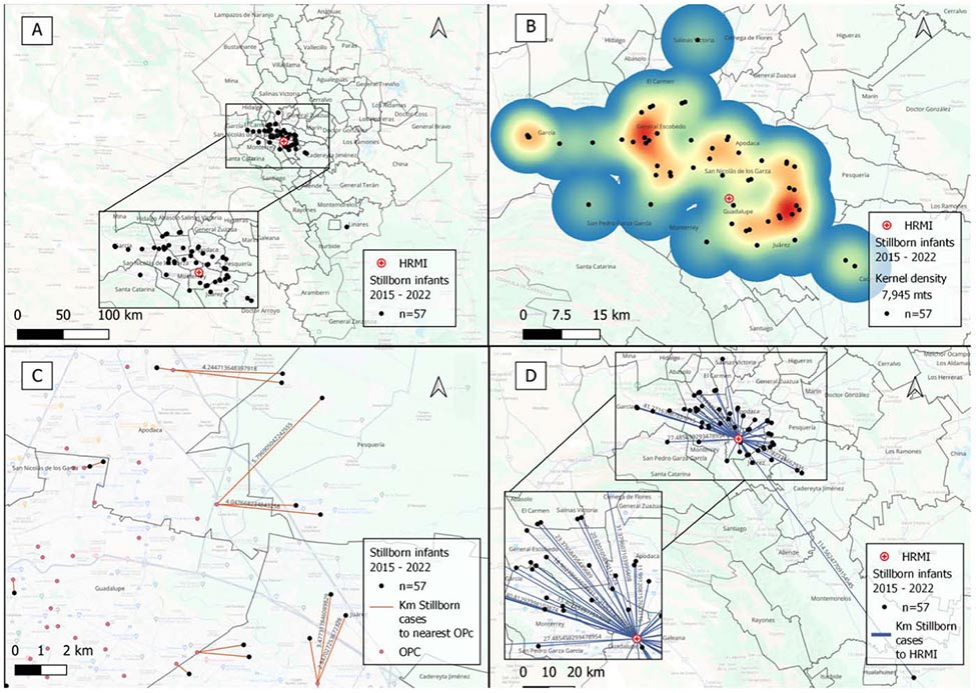
Gepgraphic and distance analysis of stillborn infants cases with syphilis infection from 2015 to 2022. A. Geographic distribution map of stillborn infants with syphilis from 2015 to 2022. B. Heat map of stillborn infants with syphilis from 2015 to 2022. C. Distance to the nearest OPC from the address of stillborn infants with syphilis from 2015 to 2022. D. Distance to HRMI from the address of stillborn infants with syphilis from 2015 to 2022

**Image 2.**
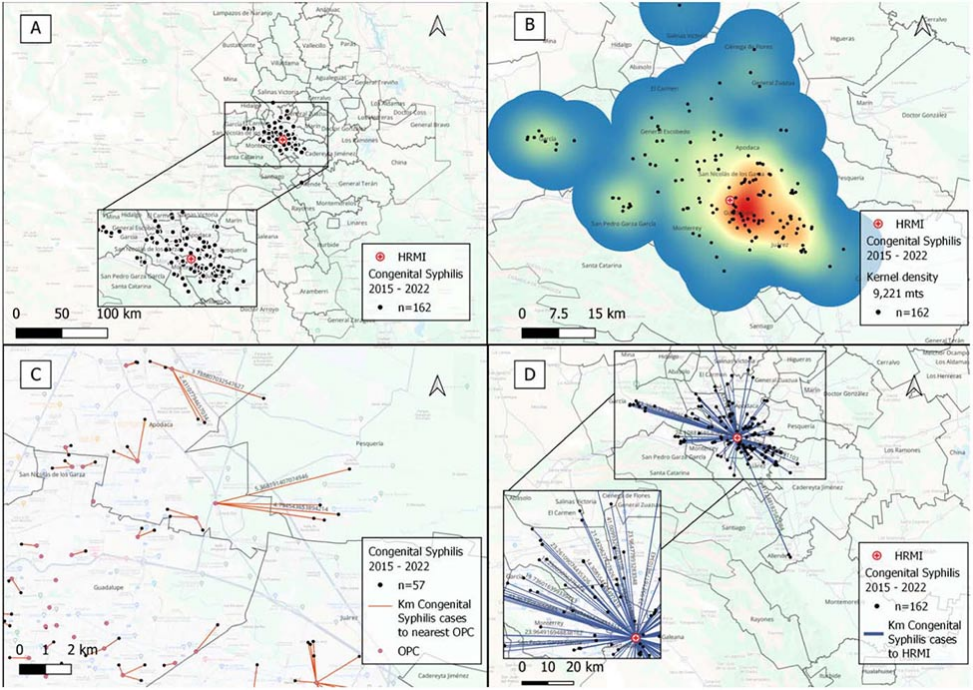
Gepgraphic and distance analysis of confirmed congenital syphilis cases from 2015 to 2022. A. Geographic distribution map of congenital syphilis cases from 2015 to 2022. B. Heat map of congenital syphilis cases from 2015 to 2022. C. Distance to the nearest OPC from the address of congenital syphilis cases from 2015 to 2022. D. Distance to HRMI from the address of congenital syphilis cases from 2015 to 2022

**Methods:**

We conducted a retrospective analysis of stillbirths associated with congenital syphilis (CS) at the Maternal and Pediatric Reference Hospital (HRMI) in Nuevo Leon, Mexico, from 2015-2022. We included data from stillborn infants (SbI) born to women with positive VDRL test at delivery, classified according to CDC criteria. Home addresses were geocoded using QGIS® (X/Y coordinates). Analyses included kernel density estimation, hierarchical clustering, and calculation of distances to the nearest outpatient clinic (OPC) and HRMI. Geographic areas were characterized using national marginalization index (NMIndx) to explore the social context surrounding each case.

**Image 3.**
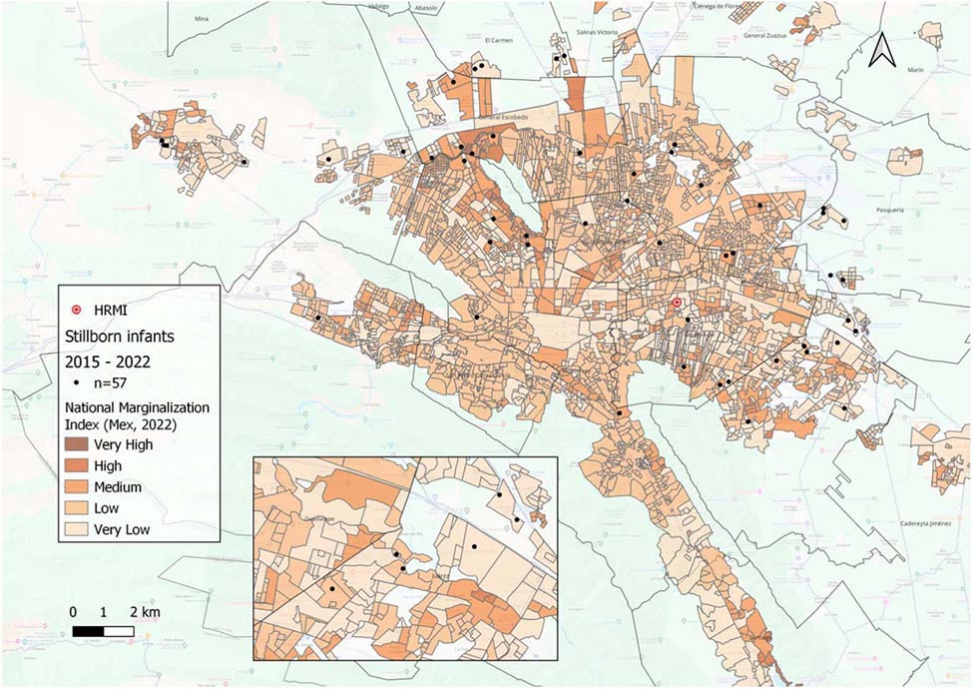
Geographic distribution and relation with National Marginalization Index (NMIndx) categorization of syphilis realted stillborn infants cases from 2015 to 2022. National Marginalization Index: Mexican government construction, official data from 2022.

**Image 3.**
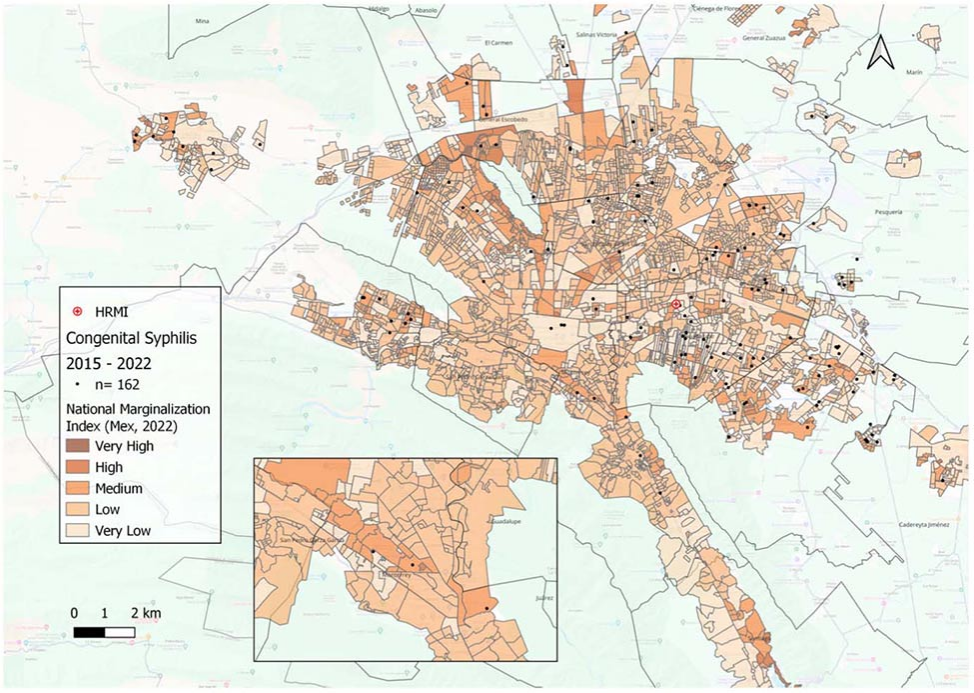
Geographic distribution and relation with National Marginalization Index (NMIndx) categorization of confirmed congenital syphilis cases from 2015 to 2022. National Marginalization Index: Mexican government construction, official data from 2022.

**Results:**

Data from 162 cases of CS and 57 SbI were included. Cases were concentrated in 3 urban municipalities. For SbI, mean distance to the nearest OPC was 1.6 km and 18 km to HRMI. For CS, 1.6 km and 14 km, respectively. Most mothers (75%) of SbI and of newborns with CS (52%) lived >10 km from HRMI. Interestingly, 71% of mothers of SbI and 77% of CS cases lived within 2 km of an OPC, where diagnosis was often missed. NNI revealed a clustering pattern with statistical significance (p < 0.05) for both SbI and CS cases throughout 8 years. Characterization of NMIndx, showed that 88% SbI and 76% CS lived in high or very high marginalization areas.

**Conclusion:**

Confirmed CS cases and SbI showed clear spatial clustering, primarily in areas with high levels of social marginalization. In contrast, low-marginalization zones reported few or no cases. These findings highlight possible geographic and social inequities driving adverse syphilis outcomes and underscore the need for targeted, community-level interventions in the most affected regions.

**Disclosures:**

All Authors: No reported disclosures

